# High Fat Diet Rapidly Suppresses B Lymphopoiesis by Disrupting the Supportive Capacity of the Bone Marrow Niche

**DOI:** 10.1371/journal.pone.0090639

**Published:** 2014-03-04

**Authors:** Benjamin J. Adler, Danielle E. Green, Gabriel M. Pagnotti, M. Ete Chan, Clinton T. Rubin

**Affiliations:** Department of Biomedical Engineering, Stony Brook University, Stony Brook, New York, United States of America; University Heart Center Freiburg, Germany

## Abstract

The bone marrow (BM) niche is the primary site of hematopoiesis, and cues from this microenvironment are critical to maintain hematopoiesis. Obesity increases lifetime susceptibility to a host of chronic diseases, and has been linked to defective leukogenesis. The pressures obesity exerts on hematopoietic tissues led us to study the effects of a high fat diet (HFD: 60% Kcal from fat) on B cell development in BM. Seven week old male C57Bl/6J mice were fed either a high fat (HFD) or regular chow (RD) diet for periods of 2 days, 1 week and 6 weeks. B-cell populations (B220+) were not altered after 2 d of HFD, within 1 w B-cell proportions were reduced by −10%, and by 6 w by −25% as compared to RD (p<0.05). BM RNA was extracted to track the expression of B-cell development markers Il-7, Ebf-1 and Pax-5. At 2 d, the expression of Il-7 and Ebf-1 were reduced by −20% (p = 0.08) and −11% (p = 0.06) whereas Pax-5 was not significantly impacted. At one week, however, the expressions of Il-7, Ebf-1, and Pax-5 in HFD mice fell by -19%, −20% and −16%, and by six weeks were further reduced to −23%, −29% and −34% as compared to RD (p<0.05 for all), a suppression paralleled by a +363% increase in adipose encroachment within the marrow space (p<0.01). Il-7 is a critical factor in the early B-cell lineage which is secreted by supportive cells in the BM niche, and is necessary for B-cell commitment. These data indicate that BM Il-7 expression, and by extension B-cell differentiation, are rapidly impaired by HFD. The trend towards suppressed expression of Il-7 following only 2 d of HFD demonstrates how susceptible the BM niche, and the cells which rely on it, are to diet, which ultimately could contribute to disease susceptibility in metabolic disorders such as obesity.

## Introduction

Hematopoietic stem cells (HSC), which reside in the bone marrow, are the source for all red blood cells, platelets and leukocytes. Leukocytes, generally described as arising from two distinct hematopoietic lineages, myeloid or lymphoid, orchestrate the body's defenses to disease and infection. HSCs ensure stable hematopoietic activity as well as the ability to respond to acute challenges to the organism [1], [2]. Hematopoietic insults, such as bleeding or irradiation, are followed by the rapid expansion of progenitor cells to repair damaged populations, replace blood, and provide the appropriate immunologic response to combat infection [3]. Indeed, the rapid expansion of progenitors following hematopoietic injury represents a critical step in restoring a fully functioning immune system, by reestablishing depleted or unbalanced cell compartments.

To a large degree, hematopoietic capacity and responsiveness is dependent on the status of the bone marrow microenvironment, which provides metabolic, physiologic and regulatory cues to the HSC, balancing quiescence and differentiation [4]. HSCs in the bone marrow microniche are co-localized, interact with, and influenced by a host of neighboring cells, including osteoblasts, osteoclasts, adipocytes and bone marrow stromal cells (BMSC), as well as adjacent tissues such as vasculature and bone trabecular and cortical surfaces [2], [5], [6]. In addition to primitive HSCs, various hematopoietic progeny which develop in the BM are also influenced in their maturation by environmental, physical and chemical signals from the niche. B-cell development in particular requires signals derived from bone marrow stromal cells and osteoblasts, among others, such as Il-7 which is critical for B-cell lineage commitment [7], [8]. Given the sensitivity of these immune cells to environmental cues, it should not be surprising that factors which compromise bone marrow composition may disrupt normal production of leukocyte populations.

Obesity increases the lifetime risk to a range of chronic illnesses [9], susceptibility fostered by a depressed and unresponsive immune system [10]. Considering that, over time, obesity causes an age-like shift of the bone marrow towards adiposity, it is possible that this encroaching fat phenotype influences the fate selection of progenitor populations and disrupts their viability [11], ultimately impairing hematopoietic reparative and regenerative potential [12], [13]. Various reports have already indicated that the obesity caused by long term exposure to high fat diet (HFD) will disrupt the normal production of leukocytes [14], [15], [16]. Nevertheless, it is not yet known how quickly this might occur, and if the diet itself contributes to the disruption of leukogenesis, or if it is ‘simply’ a byproduct of the increased adipose burden. Considering the critical role the niche plays in leukogenesis and the vulnerability of the marrow microenvironment to obesity, we hypothesize that obesity induced leukocyte defects originate very early in the process, with changes to the chemical and morphologic state of the bone marrow arising well before the obese phenotype is evident. The study reported here is designed to establish the rapidity in which a high fat diet disrupts the chemical and physical state of the bone marrow niche, and the degree to which this impacts the development and balance of immune cell populations.

## Materials and Methods

### Animals and Experimental Design

All animal protocols were reviewed and approved by Stony Brook University's IACUC. Male, C57BL/6J mice were obtained from The Jackson Laboratory (Bar Harbor, ME) at five weeks of age and housed in a dedicated animal facility. At seven weeks of age, weight matching was used to evenly distribute animals into either Regular Diet (RD), or High Fat (HF) groups. At baseline, mice were fed *ad libitum* either a standard rodent diet chow (RDC; 14% kcal from fat, LabDiet, ProLab RMH 3000), or a high fat diet (HFD; 60% kcal from fat, TestDiet, “Van Heek” 58Y1). Three time points were examined in this study with a cohort of each group being sacrificed at 2 days, 1 week, and 6 weeks after the study start. In a separate study to determine the impact of expanding adipose tissue on circulating immune populations, we investigated changes to leukocytes residing in adipose tissues following 3 weeks of HFD. All assays had a sample size of n = 8–10.

### μCT Imaging for the Assessment of Abdominal Adiposity

At the end of the six week protocol, animals were anesthetized with isoflurane inhalation and scanned *in vivo* using high-resolution micro-computed tomography μCT (VivaCT 40, Scanco, Bassersdorf, Switzerland). An abdominal region of interest spanning the L1–L5 vertebral levels was scanned at an isotropic resolution of 76 µm (45 kV, 133 µA, 300 ms integration time) [17]. A custom-designed image analysis program was used to segment adipose from other tissues, as well as delineate the visceral and subcutaneous adipose compartments [18].

### Flow Cytometric Characterization of Hematopoiesis

At sacrifice, animals were anesthetized with isoflurane inhalation and peripheral blood was drawn via cardiac puncture. After euthanasia, bone marrow was extracted from the left femur and tibia. Following erythrocyte lysis (1X Pharmlyse, BD Biosciences, San Jose, CA), cellularity was determined using an automated handheld cytometer (Scepter, Millipore, Billerica, MA). Single-cell suspensions containing 2×10^6^ cells were stained to identify populations enriched for HSCs in bone marrow, hematopoietic progenitors in bone marrow, and leukocytes including B, T, and myeloid lineage cells in bone marrow and peripheral blood.

Hematopoietic progenitors were identified based on the expression of the canonical HSC phenotype of c-Kit^Hi^-Lin^Low^-Sca-1^Hi^ (KLS). Cells expressing KLS in conjunction with the side population phenotype (SP-KLS) were considered to be HSCs [19]. Side population staining was performed according to previous protocols using a violet analogue for the classic Hoechst 33342 dye (Vybrant DyeCycle Violet, Invitrogen, Carlsbad, CA: V35003) [20]. SP-KLS cells are enriched for HSC activity in comparison to KLS cells which, although enriched for HSCs themselves, contain a significant proportion of progenitors [21].

Circulating (peripheral blood) and bone marrow resident leukocytes were stained with a protocol to identify B, T, and myeloid lineage cells on a single plot [21]. T-cells were stained with CD4 and CD8, and myeloid cells were stained with Mac-1 and Gr-1. B-cells were identified based on their expression of B220. It is important to note that this analysis did not characterize all hematopoietic lineages as, for example, it did not include either megakaryocytes or erythroblasts. Light scatter was used in all cases to exclude debris from the analysis. All antibodies were purchased from BD Biosciences. We report both the phenotypic proportion of a measured population, and an extrapolation of that population's total size.

### Characterization of BM Differentiation via RT-PCR

At sacrifice, whole bone marrow was extracted from the right femur and stored in RNAlater (Ambion, Life Technologies, Grand Island, NY). In order to extract RNA, aliquots of each sample were selected and the RNAlater was carefully removed by centrifugation. Cells were lysed using TRIzol (Invitrogen, Life Technologies), and chloroform separation was used to isolate RNA in an aqueous phase. RNA was purified using a Qiagen RNeasy kit per manufacturer's instructions, including digestion of contaminating DNA with DNase (Germantown, MD). The concentration and quality of each RNA sample was measured with a Nanodrop ND-1000 spectrophotometer (Thermo Scientific, Wilmington, DE). All reactions were performed using a StepOnePlus RT-PCR system (Applied Biosystems, Life Technologies, Carlsbad, CA). cDNAs were transcribed from each sample using Applied Biosystem's High Capacity cDNA Reverse Transcription kit (#4368814), and diluted down to a concentration of 1.5 ng/ul, based on the concentrations measured by the nanodrop device, and assuming a 1∶1 conversion ratio. Taqman gene expression assays from Applied Biosystems were used to messure the expression of Il-7 (Mm01295803_m1), Ebf-1 (Mm00395519_m1), Pax-5 (Mm00435501_m1), Runx1 (Mm01213405_m1), Crebbp (Mm01342452_m1), CXCL-12 (Mm00445553_m1), and Angiopoietin-1 (Mm00456503_m1) with mouse Actin beta (4352341E) used as a housekeeping gene. Fold change was calculated using the ΔΔC_T_ method.

### Characterization of Bone Marrow Adiposity

After six weeks of protocol, right tibiae were fixed in neutral buffered formalin for 24 hours and then stored in 70% ethanol. Bones were dehydrated with increasing concentrations of isopropyl alcohol and then embedded in polymethyl methacrylate. Thin sections (8 µm) were cut and stained for contrast using Wright-Giemsa stain (n = 6). Adipocyte ghosts were imaged using an inverted microscope (Zeiss) at 10× magnification. Marrow adiposity was quantified in the proximal metaphysis using ImageJ (NIH) to calculate total marrow area relative to the rest of the bone marrow.

To confirm the results of histology, femoral bone marrow stored in RNAlater (for PCR, see above) was assayed for triglyceride content. After homogenization, 1 ml of each sample was mixed in three volumes of a chloroform/methanol solution at a concentration of 3∶1 chloroform:methanol. After mixing and centrifugation, a portion (1 ml) of the lipid containing phase was aspirated and allowed to evaporate overnight. Following resuspension of the lipid in isopropyl alcohol (150 µl total volume), a modified protocol was used to determine the triglyceride content of the marrow in duplicate (30 µl per sample per replicate) (Serum Triglyceride Determination Kit, Sigma, St. Louis, Mo.).

### Determination of Leukocyte Subsets within Adipose Tissue by Flow Cytometry

In order to determine the impact of expanding adipose tissue on circulating immune populations, we investigated changes to leukocytes residing in adipose tissues as caused by obesity (3 W of HFD). Epidydimal fat collected at sacrifice was stored in supplemented media on ice until processing (IMDM, 2% FBS, 1% Pen/Strep, Gibco). For each sample, the left fat pad was incubated in collagenase Type II for 20 minutes at 37°C. The contents of the reaction including the remainder of the fat pad and supernatant were passed though a 40 µm cell strainer (BD Falcon, Franklin Lakes, NJ) and gently ground using the plunger of a syringe. Both the syringe and filter were washed using more supplemented media. Following centrifugation (2000 RPM, 5 minutes), red cells were lysed using 1X Pharmlyse (BD Biosciences, San Jose, CA). Single cell suspensions were then stained to indentify macrophages (F4/80) and immature myeloid cells (F4/80, Gr1, Mac1). B-cells were identified based on B220 expression and T-cells were identified based on CD8 expression. All antibodies were purchased from BD Biosciences.

### Statistics

All data are presented as means ± SD. Student's t-tests were used to test differences between groups at each time point. All statistical tests were performed using SPSS software (IBM, Somers, NY) assuming a significance level of *P*<0.05.

## Results

### High Fat Feeding Rapidly Induces the Obese Phenotype

**Figure 1:**
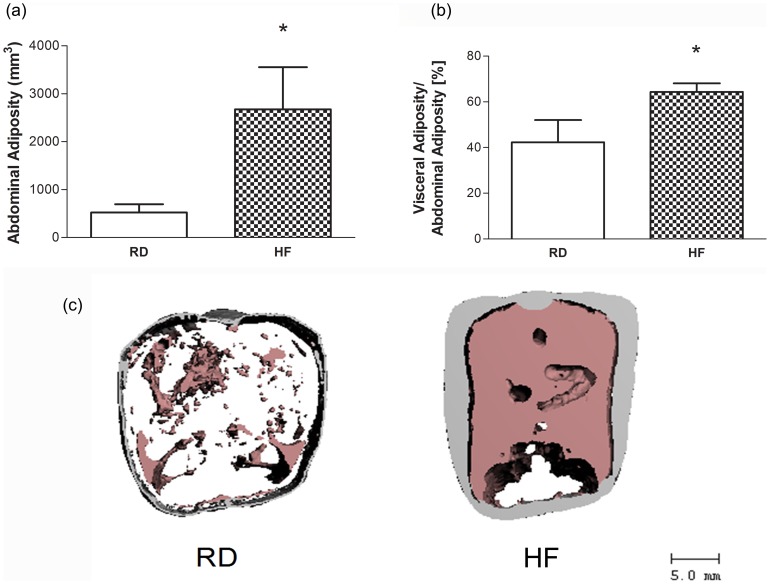
High Fat Diet Induces an Obese Phenotype. (a,b) At 6 weeks HF animals have expanded total abdominal adiposity with a prominent shift in the location of this adiposity from primarily subcutaneous to the visceral compartment. (c) The expansion and relocation of adiposity is illustrated in 3D reconstructions of abdominal μCT images which have been thresholded for adiposity (n = 9). In these images gray represents subcutaneous fat and pink represents visceral fat. *P<0.05 vs. RD.

Two days of HFD resulted in a +4% increase in body weight (n.s.) and a +28% increase in epididymal fat pad mass compared to RD (*P*<0.01; Table 1). One week on the HFD regimen increased body mass by +9% (*P*<0.05), and increased epididymal fat mass by +118% (*P*<0.01) as compared to RD. After six weeks, HF animals were +23% heavier (*P*<0.01), and epididymal fat mass was +300% greater than that of RD mice (*P*<0.01). After 6 w of HFD, total abdominal adiposity was +411% greater than that measured in RD (*P*<0.01; Fig. 1).

**Table 1:** High Fat diet leads to a significant expansion of epidydimal fat after 1 and 6 weeks compared to RD controls, demonstrating the rapid accretion of adiposity in mice fed this diet.

Epididymal Fat Weight (g)	RD	HF
**2 Days**	0.30±0.06	*0.38*±*0.06* *
**1 Week**	0.30±0.06	*0.65*±*0.18* *
**6 Week**	0.34±0.09	*1.36*±*0.46* *

*P<0.05 vs. RD.

Both visceral and subcutaneous adiposity increased over the six week HFD protocol, with the greatest increases being measured in the visceral compartment. As compared with a ratio of 42±9.9% visceral fat to total adiposity in RD, this ratio increased significantly in HF mice to 64±3.9%, representing a marked shift of adiposity to the visceral compartment (*P*<0.01; Fig. 1).

### Leukocyte Infiltration into Adipose Tissue Increases Following Shift to High Fat Diet

The onset of obesity engenders an influx of leukocytes into adipose tissue, which are central to adipose remodeling and the inflammatory cascade which parallels obesity. As measured at 3 W, HFD led to a significant invasion of leukocytes into epidydimal fat, as reflected by a +80% higher population of macrophages in HF mice as compared to control (*P*<0.01). High fat feeding also increased the presence of immature myeloid cells by +20% (*P*<0.05), while CD8 T-cells increased +60% (*P*<0.01). Interestingly, the proportion of B-cells had not increased by 3W of HFD, as HF had −6% fewer B-cells than LF mice (n.s.).

### Six Weeks of HFD Increases Marrow Adiposity

**Figure 2:**
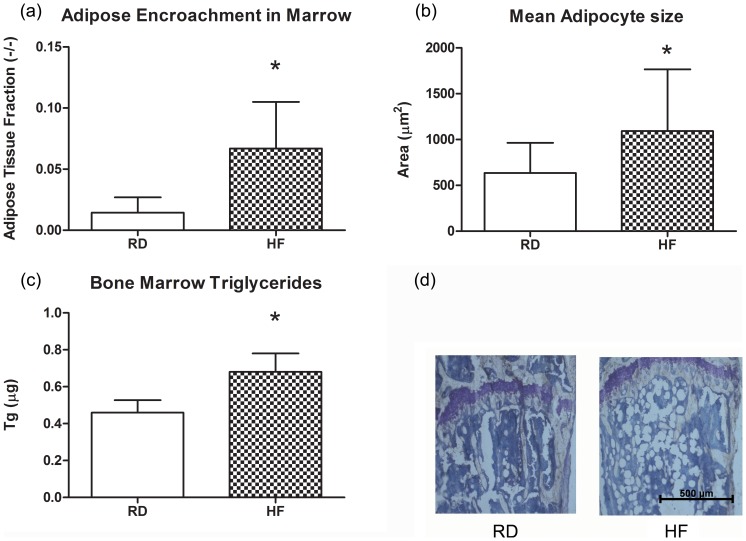
Six Weeks of HFD Rapidly Remodels the Bone Marrow Towards Adiposity. (a,b,d) The percentage of BM space taken up by adipose tissue, as assessed by histology, increased by almost 4 fold in HFD animals compared to control, an increase at least partially due to increasing adipocyte size (n = 6). (c) This leads to an increase in BM triglyceride storage (n = 10). The inclusion of adipocytes in the BM is significant because of the deleterious paracrine and inflammatory consequences adipocytes are thought to present to hematopoiesis. Additionally, BM adiposity in general represents a decline in BM quality and health, as is demonstrated by increased BM adiposity in osteoporosis and anorexia nervosa. *P<0.05 vs. RD.

Following six weeks of HFD, the adipose tissue fraction of BM increased by +363% compared to RD (*P*<0.01). Similarly, the average adipocyte area, measuring 636±328 µm^2^ in RD mice, increased +72% in HF to 1093±672 µm^2^ *P*<0.01;Fig. 2). At this same time point, triglycerides measured in the bone marrow increased by +48% in HF mice as compared to RD controls (*P*<0.01).

### High Fat Diet Disrupts Hematopoietic Lineage Allocation

**Figure 3:**
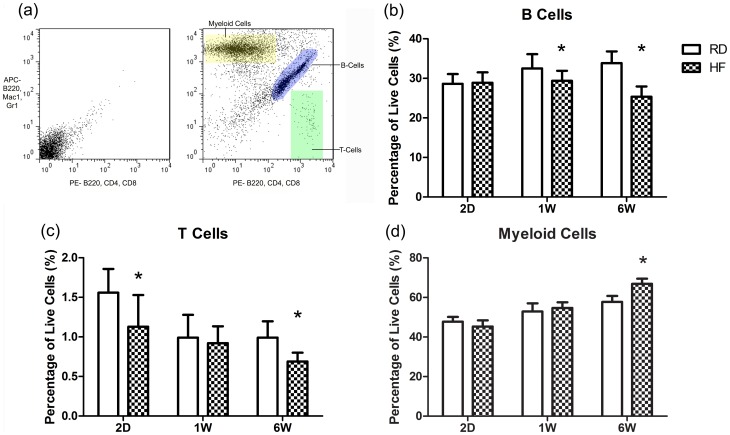
High Fat Diet Initiates an Aged Leukocyte Phenotype in the Bone Marrow. (a) A two-color flow cytometric method was used to isolate leukocyte populations of the lymphocyte (B and T-cells) and myeloid lineages, with the unstained control displayed in the left panel. (b–d) At two days a T-lymphopenia was present. At one week of HFD B-cell populations were diminished in the HF group, while T-cell populations were not significantly affected. After six weeks bone marrow lymphocyte populations were depressed and the myeloid lineage proliferated in response to HFD. The suppression of lymphocytes accompanied by myeloid expansion is a classic phenotype of hematopoietic aging. These changes may lead to defects in adaptive immunity. Each time point was drawn from distinct cohorts of animals, as such, comparisons can only be made within a time point due to variability in processing. *P<0.05 vs. RD.

Leukocytes isolated from the peripheral blood and bone marrow were used for analysis of B-cells, T-cells and myeloid lineages (Fig. 3). HFD had no impact on the level of circulating leukocytes in the peripheral blood at any time point studied here, and preliminary data indicates that enhanced extramedullary hematopoiesis may have played a role in maintaining these circulating populations (data not shown). In contrast, the proportions of leukocytes in the bone marrow were significantly compromised by the high fat diet regimen. After 2 d, HF animals had −28% fewer T-cells than RD (*P*<0.05), and −5% fewer myeloid cells (*P* = 0.07) in the bone marrow, with no differences seen between HF and RD B-cell populations at this time point. After 1 w, however, the impact of HFD on leukocyte populations had reversed, with mice fed a high fat diet displaying decreased B-cells (−10%, *P*<0.05), but no differences measured between HF and RD in either myeloid or T-cells.

By six weeks, the bone marrow displayed an “aged” phenotype in HF animals [22] with the proportion of T-cells falling by −31%, and B-cells down by −25% as compared to RD (*P*<0.01), while the myeloid cell fraction rose by +16% (*P*<0.01; Fig. 3). These data emphasize the vulnerability of hematopoietic lineages to HFD even within a very short time period, and that this disruption persists over time.

**Figure 4:**
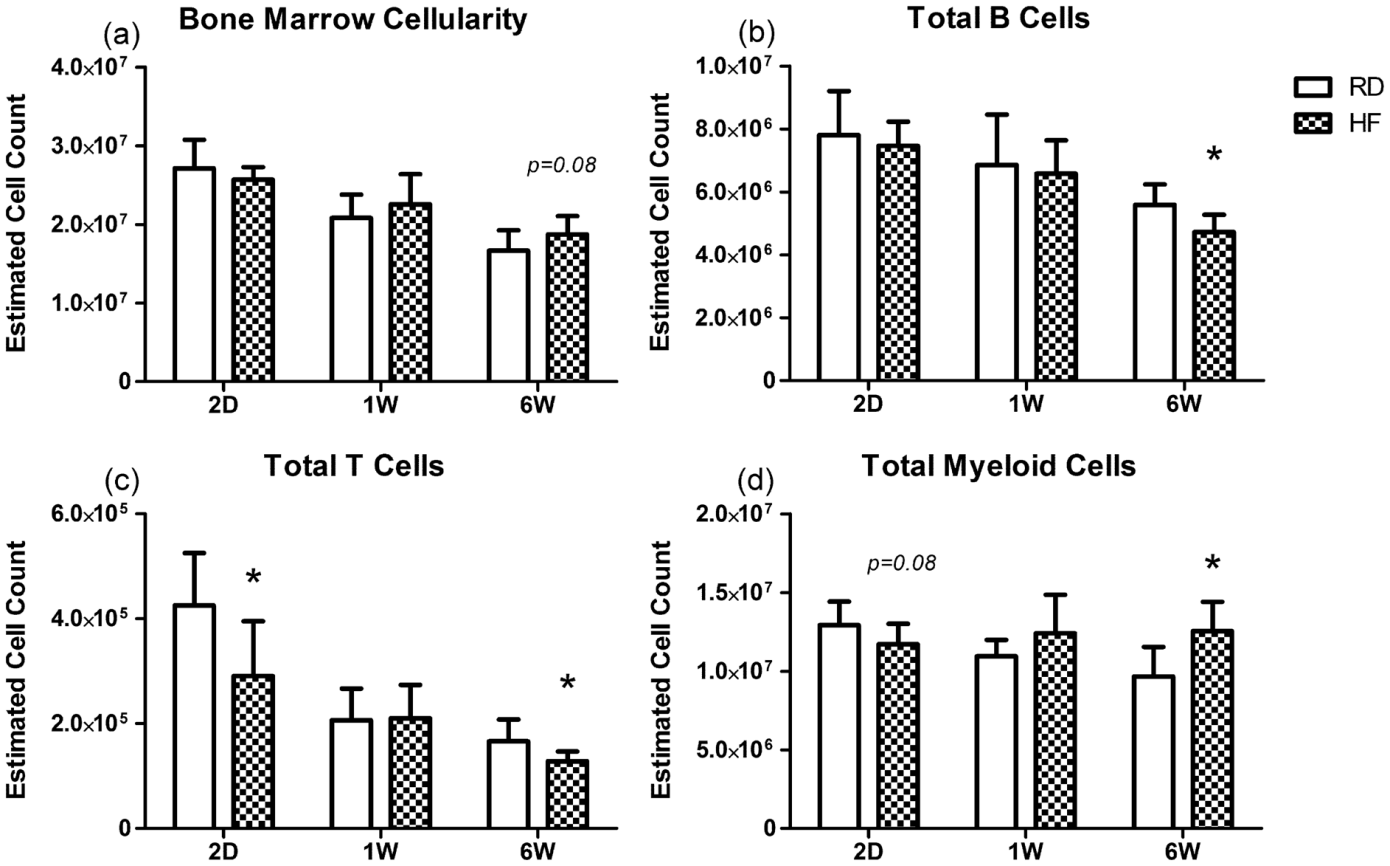
High Fat Diet Alters the Total Population Sizes of BM Leukocytes. (a) Total BM cells harvested for flow cytometry were not different between HF and RD animals at any time point, but a trend of increase was observed at 6W. (b) The total number of B-cells was not changed at 2D or 1W contrary to a decline in the phenotypic population at 1W. However, B-cells were reduced at 6W, despite a slight increase in total cellularity, demonstrating the degree of population reduction. (c,d) Total population sizes of both T-cells and Myeloid lineages tracked well with the phenotypic population proportions. *P<0.05 vs. RD.

In order to understand if leukocyte populations were truly changing, or merely changing with respect to one another, we also extrapolated to the total population sizes by multiplying the measured proportion by the total number of cells harvested for flow cytometry. Total cellularity was not different between the groups at any time point, however there was a trend of increased cell content at 6 W in the HF animals (+13%, *P* = 0.08). The total number of T-cells tracked very well with their phenotypic proportions being −32% and −30% lower in HF than RD animals at 2D and 6W (*P*<0.05), and not different from RD at the 1W time point (+2%, n.s.). Myeloid population sizes also closely matched the reported proportions, with −9% fewer myeloid cells in HF animals after 2D (*P* = 0.08), a non-significant +13% increase at 1W, and a +16% increase after 6W of HFD (*P*<0.01). Interestingly B-cell population sizes bucked the trend of their proportional counterparts at the 1W time point. Whereas proportionally there were −10% fewer B-cells in HF animals at 1W, in estimated total numbers this decrease was only −4% (n.s.). Both the 2D and 6W time points did correspond to the proportional data, as a deficit of total B-cells emerged at the 6W time point (−25%, *P*<0.01)(Fig. 4).

### Transient Hematopoietic Response to HFD

To determine if the HFD mediated disruption of leukocyte allocations were paralleled by changes to the primitive hematopoietic system, HSCs and their earliest progeny were isolated from the bone marrow. No HFD mediated differences were observed in the primitive SP-KLS population at any time point, perhaps an indication of these cells' quiescence, especially over a short term of exposure to HFD.

**Figure 5:**
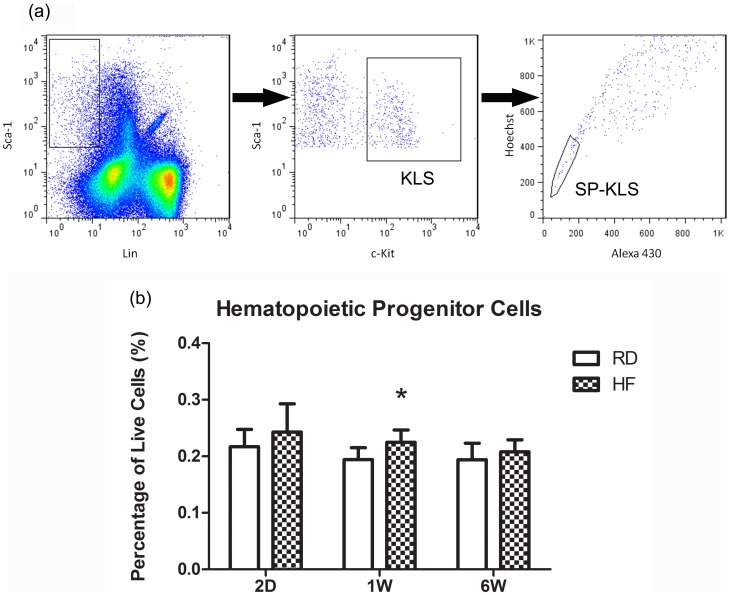
Hematopoietic Progenitors Rapidly Respond to High Fat Diet Mediated Damage. (a) A representative gating scheme to isolate KLS and SP-KLS cells is shown. HSCs (SP-KLS) were assessed to determine the hematopoietic response to obesity driven leukocyte mis-allocation but no differences were found at any time point studied. (b) Hematopoietic progenitors rapidly respond to obesity mediated damage displaying an early response to HFD with HF animals having elevated KLS populations at 1 week. By 6 weeks this response was no longer evident in HF animals. *P<0.05 vs. RD.

While HSCs were not significantly impacted by diet, HFD did influence the more differentiated hematopoietic progenitor cells (KLS). After 2 d, this population was non-significantly elevated in HF by +15%, while within one week the KLS cell fraction increased by +16% relative to RD (*P*<0.01; Fig. 5). This increase in hematopoietic progenitors may be indicative of an early strategy to restore and rebalance leukocyte differentiation as disrupted by HFD. However, this reparative response measured in the HF mice was transient, as the +7% greater number of progenitor cells measured at 6 weeks was not significantly different from that seen in RD.

### HFD Developmentally Limits Bone Marrow Lymphopoiesis

**Figure 6:**
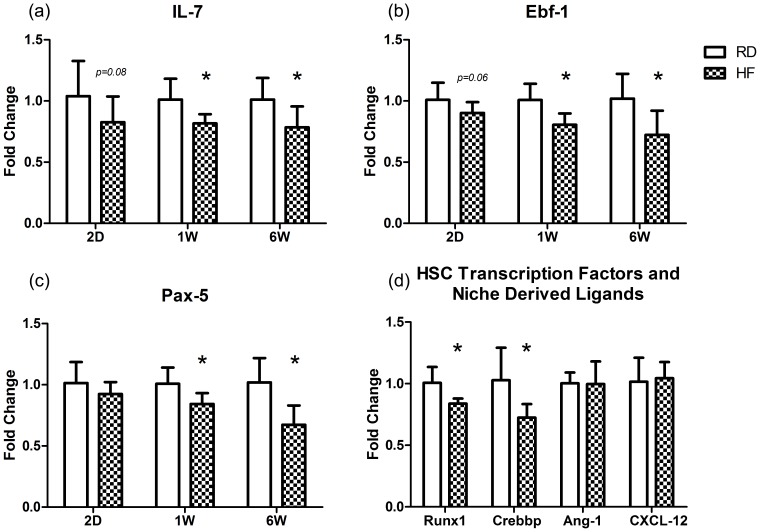
HFD Developmentally Decreases B Lymphopoiesis: RT-PCR was performed on whole BM extracted from the femur. (a,b) Il-7 and Ebf-1, early markers of lymphoid commitment showed strong trends of reduced expression after only 2D of HFD and were significantly reduced in HF animals from 1W onward. (c) Pax-5 expression, which represents an intermediate step in B lymphopoiesis did not appear reduced at 2D, but was significantly decreased subsequently. (d) Runx1 and Crebbp, which are critical regulators of hematopoiesis, were down regulated following 6 weeks of HFD demonstrating that obesity impacts the commitment and differentiation leukocytes as well as HSCs themselves. However, the expression of CXCL-12 and Angiopoietin-1 were not influenced by obesity (6W), demonstrating that the impact of obesity on IL-7 signaling is specific. *P<0.05 vs. RD.

To examine the origins of the lymphopenic phenotype, RT-PCR was performed on whole bone marrow extracted from femurs at 2D, 1W, and 6W (Fig. 6). After 2 d of HFD, the expressions of Il-7 and Ebf-1, critical to early B-cell development, were down −20% (*P* = 0.08) and −11% (*P* = 0.06), while Pax-5 expression, a marker indicating an intermediate stage of development, showed only a slight trend in reduction relative to RD (−9%; p = 0.18). At 1 w, the expression of all three genes was suppressed in the HF mice, with Il-7 decreased by -19%, Ebf-1 reduced by −20%, and Pax-5 down by −16% (*P*<0.01 in each) relative to RD. These relative reductions persisted at 6 w, with the expression of Il-7, Ebf-1, and Pax-5 down-regulated by −23%, −29%, and −34%, respectively (*P*<0.01). Runx1 and Crebbp expression were decreased after 6 w in HF by −17% and −24%, as compared to RD (*P*<0.05). The expressions of CXCL-12 and Angiopoietin-1 were not changed at either 2D or 6W.

## Discussion

Obesity fosters a host of chronic, systemic health complications which conspire towards a marked decrease in life expectancy [23]. That obesity also increases the risk of infection and illness [24], [25], implicates that elevated adiposity ultimately impairs immunity. In the work presented here, we examined the possibility that a high fat diet could rapidly disrupt hematopoiesis even during the earliest stages of the initiation of obesity.

It is well recognized that obese mice present with diminished immune cell function [16], [26]. In addition to the impairment of leukocyte function *per se*, obesity weakens the health of hematopoietic tissues and the maintenance of appropriate population sizes of leukocytes. Indeed, Yang et al. showed that frank obesity as caused by 11 months of a HFD was correlated to severely compromised lymphoid lineages in the bone marrow, leading to thymic involution and defective T-cell production [27]. Additionally, obesity as caused by seven months of a HFD (45% kcal from fat) was associated with B lymphopenia amongst both developing (BM) and circulating cells, as well as significant trabecular bone loss, an indication of deleterious trends in the BM niche [14]. Interestingly, only circulating T-cells, and not those in the bone marrow, were disrupted by HFD, suggesting that T-cell populations are less indicative of the health of the bone marrow than B lymphocytes; at least when impacted by changes in diet. The central role that BM plays in the genesis and development of leukocytes led us to hypothesize that any impact of a high fat diet - as a prequel to obesity - on immune populations might be initiated by early disruption of the BM environment and thus the cells that reside in it. In this study, we set out to trace the origins of such lymphopenic phenotypes both in terms of initial occurrence and their relationship to changes in the niche.

The impact of high fat diet on lymphopoiesis was seen as early as two days, as evidenced by a subtle decline in BM T-cell populations. T-cells recovered to normal population levels by 1w, but regressed again to levels lower than RD controls at 6w. Whereas previous reports have linked long term T lymphopenia in obesity to developmental issues in the thymus [27], the early decline and then recovery of BM T-cells observed here suggests that, at least initially, T-cell production is not impaired by obesity *per se*, but rather these populations may be recruited elsewhere in response to a systemic increase in the adipose burden initiated by the high fat diet. T-cell populations have been suggested to precede macrophage infiltration of visceral adipose tissue, helping to initiate the inflammatory cascade in rapidly expanding adipose depots [28]. That these fluctuations happen so quickly may imply an insult caused by diet as much as by the change in the adipose phenotype, and in reality, they may both contribute to the change in T-cell levels. Indeed, as evidenced by data from the 3W protocol, the obese mice showed a significant infiltration of CD8 T-cells into the adipose tissue. Additionally, populations of macrophages and immature myeloid cells increased significantly in the fat pads. Similar data has been shown previously elsewhere, as already indicated, however in this case these data are used to illustrate the rapid systemic effects of high fat diet. Specifically, short durations of high fat diet strongly influence inflammation and leukocyte populations and trafficking in diverse anatomical sites, even in the absence of an impact on circulating populations.

Unlike T-cells, which exit the bone marrow at a relatively early stage of development to mature in the thymus, B-cells remain in the BM niche further into their maturation. This prolonged exposure of the B-cell progenitor pool to the disrupted regulatory environment of the bone marrow invariably would make B-cell development more susceptible to the changes in the BM phenotype. For this reason, particular emphasis was placed on understanding the influence of short term HFD and obesity on the B-cell lineage.

These studies indicate that a high fat diet rapidly catalyzes a decline in B-cell populations, the origins of which appear to begin at the earliest stage of B-cell commitment. The first phenotypic indication of a B-cell defect occurred at 1w, degenerating further by 6w at which point total numbers of B-cells were also reduced. Additionally, despite the relative youth of these animals (13 weeks at time of sacrifice), the overall bone marrow phenotype displayed classic signs of age-related dysfunction, characterized by myelo-proliferation and diminished lymphopoiesis [22]. This rapid disruption of the bone marrow phenotype demonstrates both local and systemic consequences of a high fat diet, and its associated adiposity, and its capacity to harm leukogenesis.

Supporting a conclusion that the high fat diet disrupts fate selection in the earliest stage of HSC progenitors, RT-PCR was used to show changes in a range of genes that indicated that the depletion of B-cells could be traced back along the differentiation pathway to even the earliest points in commitment to the lineage. For example, Ebf-1 is recognized as an important regulatory and survival factor in the earliest stages of B lymphopoiesis [29], and is required for lineage commitment as well as the successful transition from the pro- to pre-B stage of development [30]. The initiation of Ebf-1 expression has been recently linked to a functional switch within the CLP compartment associated with B-cell lineage restriction. This switch is dependent on Il-7, a cytokine produced by hematopoietic stroma [8]. Our data demonstrated strong trends of reduced Il-7 and Ebf-1 expression after only 2d of HFD, prior to a significant reduction of Pax-5 expression or any reduction of B220+ B-cells. Following 1w of HFD, Il-7, Ebf-1 and Pax-5 were all significantly down regulated, correlating well with an emerging depletion of phenotypic B-cells. However, despite the reduction in the phenotypic B-cell proportions, total numbers of B-cells were not reduced until the 6W time point. This fact combined with the early down regulation of B-cell differentiation factors points to the developmental nature of the B-cell depletion, originating in diminished maturation of primitive B-cells. Continued exposure to a high fat diet through six weeks led to further suppression of B lymphopoiesis at each stage examined, from lineage restriction (Il-7 and Ebf-1), through intermediate developmental stages (Pax-5) including a robust suppression of phenotypic B-cells (B220+), ultimately resulting in the aforementioned reduction in population size. Therefore, we conclude that a high fat diet induces B lymphopenia by developmentally limiting the production of B-cells.

The early down regulation of Il-7 and Ebf-1, at the head of the B lineage, signifies that the change to a high fat diet disrupts leukogenesis at early stages of commitment, in this case by depriving progenitors of critical environmentally derived differentiation cues. We hypothesize that it is the reduction in Il-7 expression which initiates lymphopenia by restricting lineage commitment, indicating that changes to the BM niche are responsible for the hematopoietic disruption observed in these studies. The ongoing reduction of Il-7 may further amplify the lymphopenic phenotype by harming the survival of Il-7r+ cells, many of which are committed B progenitors [8]. Recent work has implicated IL-7 production by osteoblasts as being critical to B-lymphopoiesis [31]. Osteoblasts have long been known to play important roles in the hematopoietic niche, particularly in regulating B-cells [32], [33]. These data suggest an influence of obesity on osteoblasts, however more work is needed to clarify the mechanisms through which systemic or local adiposity might diminish osteoblastic IL-7 production. Interestingly, the expressions of CXCL-12 and Angiopoietin-1 were not reduced by the onset of obesity. These genes encode proteins which, similar to IL-7, are involved in the maintenance of hematopoietic cells in niches comprised of single or few cell clusters. The fact that the expression of these genes was unaffected implies that IL-7+ osteoblasts, and not other niche cells and ligands, were impacted by obesity in a targeted, rather than generalized manner.

The susceptibility of Il-7 expression in the bone marrow, and by extension B lymphopoiesis to a high fat diet has not previously been reported, and illustrates the delicate balance within the hematopoietic niche and the populations that depend on it. These data reflect the speed with which this change to a high fat diet and concomitant obesity causes functional and phenotypic changes in the niche, and the long-term consequences to the immune system that these initiate. This conclusion is supported by the marked changes in the composition of the marrow niche, driving towards 3-fold increases in adipose burden in the marrow cavity after only 6 weeks of HFD. BM adiposity is widely thought to hamper hematopoiesis through direct paracrine and inflammatory insults [11]. What is interesting is that BM adiposity is not unique to obesity, but rather is a phenotype present in various states of impaired health, such as osteoporosis, aging, anorexia, and models of irradiation induced bone marrow failure [34], [35]. This may imply that BM adiposity, in addition to a causative role in the development of obese hematopoietic defects, may also be a concomitant result and signal of diminished BM quality.

Injury to the hematopoietic system is often paralleled by an increase in hematopoietic progenitor (KLS cell) proliferation, a response interpreted as a central reparative strategy to restore balanced hematopoiesis [36]. The scale of this reparative response, and the exact populations involved are suspected to vary with the nature and severity of injury [3]. We believe these data provide further evidence of the consequences of a dietary stressor to rapidly disrupt hematopoietic populations, an imbalance which in turn signals HSCs to differentiate towards hematopoietic progenitors as a step towards restoring the leukocyte reservoir.

Similar to the significant elevation of KLS populations within one week of the introduction of a high fat diet, infection with vaccinia virus has been shown to lead to a rapid (1 day) increase in bone marrow KLS cells, a response which subsides when leukocyte progenitors show evidence of normalizing to healthy levels [37]. However, the continued restriction of Il-7 expression indicates that such attempts at repair might be undermined *a priori*, unless and until the aggravating factor itself is removed. Indeed, while KLS cells in HFD displayed a significant elevation in response to diet-induced damage at one week, by six weeks this progenitor pool appears unable to sustain these elevated levels and returned to those levels measured in RD. While an extrapolation, we interpret the retreat of hematopoietic progenitor populations in the high fat diet group - despite evidence of continued leukocyte dysregulation - as an indication of a systemic collapse of the hematopoietic reparative response in spite of persistent dietary and adipose challenges. Indeed, our data also indicate a loss of transcriptional control in more primitive HSCs, as seen in the lowered expression of Runx1 and Crebbp at 6w. Both of these genes are transcription factors which play crucial roles in hematopoietic development and quiescence, and it has been postulated that their expression is antagonistic to myelopoiesis [38], [39], [40]. The decrease of expression in these genes implies a loss of HSC regulation and, perhaps ultimately, loss of function due to both a high fat diet and the obese phenotype. As both Runx1 and Crebbp have been associated with HSC regulation through intrinsic and niche mediated mechanisms, it is not clear at this stage whether this is another consequence of high fat diet initiated damage to the niche, or of direct impact on the HSCs [41], [42]. What is clear from these data is that continued exposure to a high fat diet has the potential to markedly disrupt hematopoiesis, from the earliest stages of primitive HSC lineage selection to the ultimate production and function of leukocytes.

## Conclusions

These data demonstrate that introduction to a high fat diet catalyzes a rapid disruption of the BM niche, the consequences of which include dysregulation of B-cell populations. Further, our data suggest that defects in lymphogenesis are the result of impairments to niche function early in the development process, resulting in diminished cellular differentiation. It is currently unclear if these processes are mediated primarily by obesity, or if the increase in adiposity and some combination of dietary factors are causative. Although a high fat diet also appears to cause dysregulation to more primitive HSCs, it is unclear if this damage is intrinsic, or niche mediated, and to what extent this damage is already manifesting in the development of leukocyte populations.
